# *Myceliophthora thermophila* Xyr1 is predominantly involved in xylan degradation and xylose catabolism

**DOI:** 10.1186/s13068-019-1556-y

**Published:** 2019-09-16

**Authors:** Ana Carolina dos Santos Gomes, Daniel Falkoski, Evy Battaglia, Mao Peng, Maira Nicolau de Almeida, Nancy Coconi Linares, Jean-Paul Meijnen, Jaap Visser, Ronald P. de Vries

**Affiliations:** 1 Fungal Physiology, Westerdijk Fungal Biodiversity Institute & Fungal Molecular Physiology, Utrecht University, Uppsalalaan 8, 3584 CT Utrecht, The Netherlands; 2 Present Address: Novozymes Latin America, Professor Francisco Ribeiro Street 683, Araucária, PR 83707-660 Brazil; 3 DuPont Industrial Biosciences, Archimedesweg 30, 2333 CN Leiden, The Netherlands; 4 Present Address: Federal University of São João del Rei, Praça Dom Helvécio, 74, São João del Rei, Minas Gerais Brazil; 5 Present Address: Dutch DNA Biotech BV, Padualaan 8, 3584 CH Utrecht, The Netherlands

**Keywords:** *Myceliophthora thermophila*, Xylanolytic regulator, Pentose catabolism, Xylan degradation, Cellulose degradation

## Abstract

**Background:**

*Myceliophthora thermophila* is a thermophilic ascomycete fungus that is used as a producer of enzyme cocktails used in plant biomass saccharification. Further development of this species as an industrial enzyme factory requires a detailed understanding of its regulatory systems driving the production of plant biomass-degrading enzymes. In this study, we analyzed the function of MtXlr1, an ortholog of the (hemi-)cellulolytic regulator XlnR first identified in another industrially relevant fungus, *Aspergillus niger*.

**Results:**

The *Mtxlr1* gene was deleted and the resulting strain was compared to the wild type using growth profiling and transcriptomics. The deletion strain was unable to grow on xylan and d-xylose, but showed only a small growth reduction on l-arabinose, and grew similar to the wild type on Avicel and cellulose. These results were supported by the transcriptome analyses which revealed reduction of genes encoding xylan-degrading enzymes, enzymes of the pentose catabolic pathway and putative pentose transporters. In contrast, no or minimal effects were observed for the expression of cellulolytic genes.

**Conclusions:**

*Myceliophthora thermophila* MtXlr1 controls the expression of xylanolytic genes and genes involved in pentose transport and catabolism, but has no significant effects on the production of cellulases. It therefore resembles more the role of its ortholog in *Neurospora crassa*, rather than the broader role described for this regulator in *A. niger* and *Trichoderma reesei*. By revealing the range of genes controlled by MtXlr1, our results provide the basic knowledge for targeted strain improvement by overproducing or constitutively activating this regulator, to further improve the biotechnological value of *M. thermophila*.

## Background

Plant biomass feedstocks are abundant and cheap waste materials which are increasingly used by different bio-based industries to produce a range of products. Bioethanol and biodiesel are examples of such second-generation biorefinery products which can be produced from lignocellulosic-rich biomass feedstocks [1]. These feedstocks include agricultural residues (e.g., by-products of cereal crops and sugarcane bagasse), forestry waste (e.g., wood chips and sawdust) and perennial grasses (e.g., miscanthus and switchgrass) [2, 3]. Lignocellulose is a heterogeneous and recalcitrant substrate that consists mainly of polysaccharides and lignin [4]. Its degradation requires costly processes, e.g., a thermochemical pre-treatment followed by enzymatic hydrolysis, to degrade those polysaccharides into fermentable sugars for the production of bioethanol or biochemicals [5]. Currently, there is still a need to improve available bacterial and fungal enzyme preparations [6]. To reduce the costs of the hydrolysis process, enzymes and enzyme mixtures are needed that are able to more efficiently and more completely solubilize the lignocellulosic material resulting in higher yields of fermentable sugars, above the 60% that is currently often reached.

The application of thermostable enzymes to saccharify lignocellulosic feedstocks is therefore gaining wide industrial interest [7]. Thermostable enzymes have several advantages over mesophilic enzymes, such as higher thermal stability enabling longer hydrolysis times if needed and higher specific activity at higher temperatures thus reducing the enzyme dosage needed [8]. The use of higher temperatures during saccharification (e.g., 50–60 °C) will result in better substrate solubility and lower viscosity, higher mass transfer rates and a lowered risk of contamination [9]. Therefore, applying thermostable enzymes in lignocellulose saccharification may lead to a decrease in enzyme dosage, faster conversion rates and higher sugar yields, thus reducing the overall production costs.

*Myceliophthora thermophila* is such a thermophilic ascomycete fungus, which is soilborne and also occurs in self-heated compost [10]. *M. thermophila* is known to produce a complete set of cellulolytic enzymes that act synergistically when grown on cellulose [11, 12]. A large set of lignocellulosic enzymes from *M. thermophila* has previously been biochemically characterized (reviewed in [13]). All enzymes have optimal activity at temperatures between 40 and 85 °C, and many of them display high thermostability [13–17]. Besides these characterized enzymes, the genome of *M. thermophila* contains many more genes encoding (putative) carbohydrate-active enzymes involved in oxidative and hydrolytic (hemi-)cellulose degradation. Those may also have thermophilic properties of interest for industrial applications [10, 13, 18]. This powerful potential for lignocellulose conversion has been shown when the fungus was grown on various untreated agricultural substrates [10, 19–21] and on pretreated plant biomass [19, 22]. For this reason, the industrial *M. thermophila* strain C1 was developed into an enzyme production platform, optimized for commercial-scale production of xylanolytic and cellulolytic enzymes to release both C5 and C6 sugars efficiently [23].

The production of lignocellulosic enzymes is mainly regulated at the transcriptional level in ascomycete fungi. Three transcription factors play a dominant, but not always the same role in the breakdown of cellulose and hemicellulose: Clr1 (ClrA) and Clr2 (ClrB) [24] and XlnR (Xyr1/Xlr1) [25, 26]. In this paper we focus on the xylanolytic activator XlnR (Xyr1/Xlr1), which thus far is the most extensively studied transcription factor involved in lignocellulose degradation in ascomycete filamentous fungi. XlnR/Xyr1 is involved in the regulation of both cellulolytic and hemicellulolytic genes in *A. niger*, *Aspergillus oryzae* and *T. reesei* [25, 27–29]. However, previous studies showed differences in the function of XlnR (Xyr1/Xlr1) in different filamentous fungi such as *N. crassa* and *Magnaporthe grisea* where this factor is only required for hemicellulose degradation [25, 30].

Overexpression of the *xlnR* homolog in the *M. thermophila* ATCC42464 strain (*Mtxyr1*) has been found to result in an increase in xylanase production, but not in cellulase production during growth on media containing an agricultural waste substrate (corncobs) or d-glucose [31]. Therefore, we decided to investigate the xylanolytic system and its regulation in this industrially relevant fungus in more detail to create the knowledge base required for a dedicated strain improvement program. While we have used a different strain than ATCC42464 and differences between strains cannot be excluded, both strains have been demonstrated to be closely related using phylogenetic means (de Vries et al. unpublished results).

## Results

### Deletion of *Mtxyr1* affects growth on **d-**xylose and different xylans, and uptake of **d-**xylose

The complete ORF of the *Mtxyr1* gene was deleted in *M. thermophila* C1 by targeted gene replacement via homologous recombination and verified by PCR analysis in three independent transformants (see Methods and Additional file 1: Fig. S1) with identical phenotypes. Two transformants were complemented with the wild-type gene, restoring the wild-type phenotype (Additional file 1: Fig. S2). Based on this, one of the transformants (Δ*xyr1.1C;* further referred to as *ΔMtxyr1*) was used for further analyses.

**Fig. 1:**
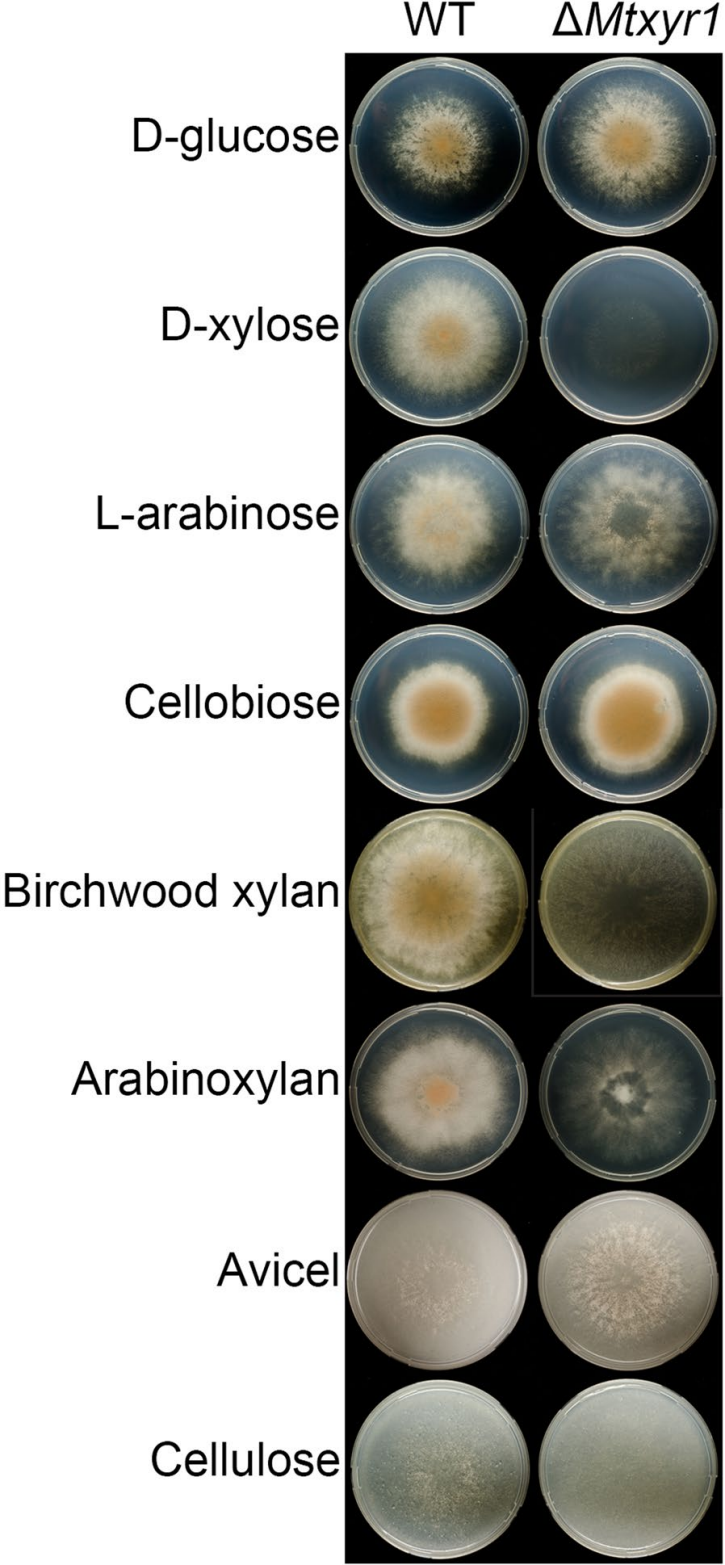
Growth phenotype of the *M. thermophila* wild type and Δ*xyr1* on agar plates. The wild-type strain and the *Mtxyr1* deletion mutant (Δ*Mtxyr1*) were grown for 5 days at 37 °C on solid minimal medium containing different mono- oligo- and polymeric carbon sources

Δ*Mtxyr1* showed hardly any growth on solid media plates with d-xylose and birchwood xylan, reduced growth on wheat arabinoxylan compared to the wild type, while growth was only slightly reduced on l-arabinose (Fig. 1). No effect on growth of Δ*Mtxyr1* was observed on d-glucose, cellobiose, Avicel and cellulose (Fig. 1).

**Fig. 2:**
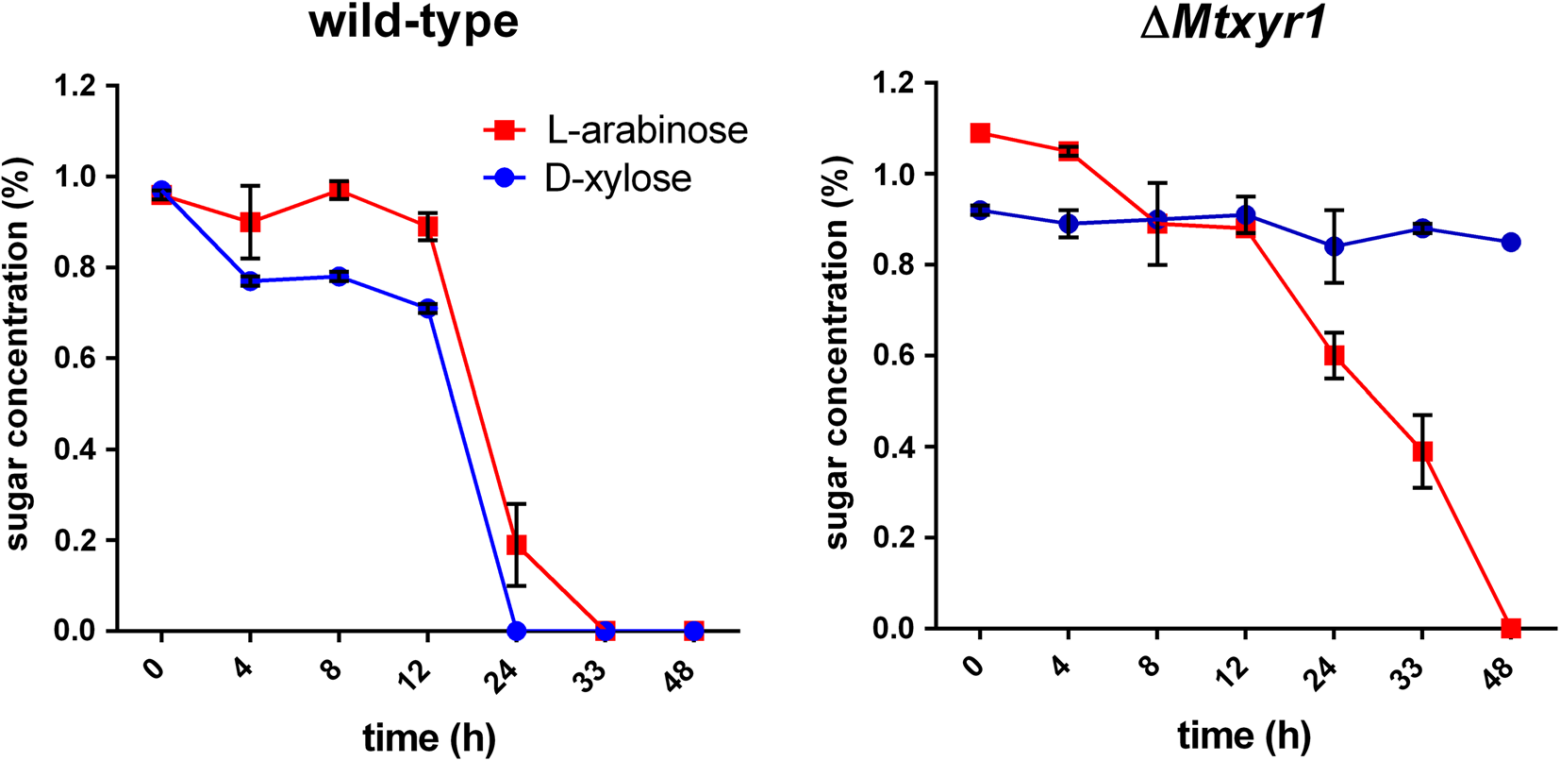
Extracellular l-arabinose and d-xylose concentration during 48 h of growth of the wild-type and Δ*Mtxyr1* strain in liquid cultures. Extracellular l-arabinose (red line) and d-xylose (blue line) concentration measurements of the wild type and Δ*Mtxyr1* grown in 50 mL liquid shake flask cultures with l-arabinose or d-xylose

To determine whether deletion of *Mtxyr1* has an effect on uptake of d-xylose and l-arabinose, the wild-type and Δ*Mtxyr1* were pre-grown in minimal medium with d-fructose for 48 h in liquid shake flask cultures and aliquots of mycelium were then transferred to shake flask cultures with l-arabinose or d-xylose (see Materials and Methods). d-xylose and l-arabinose concentrations were measured in the extracellular medium over time (Fig. 2). During cultivation of the wild type in minimal medium with d-xylose or l-arabinose alone, each sugar concentration decreased slowly during the first 12 h of growth (Fig. 2), but more rapidly during the next 12 h. d-xylose and l-arabinose were depleted after 24 h and 33 h, respectively. The d-xylose concentration in the extracellular medium of Δ*Mtxyr1* remained at a similar level during 48 h of growth (Fig. 2), while l-arabinose gradually decreased over time, yet slower than in the wild type, to be completely depleted after 48 h.

### The activity of endo-xylanases, β-xylosidases and cellobiohydrolases is reduced in the Δ*Mtxyr1* strain on **d-**xylose, **l-**arabinose and/or different xylans

**Fig. 3:**
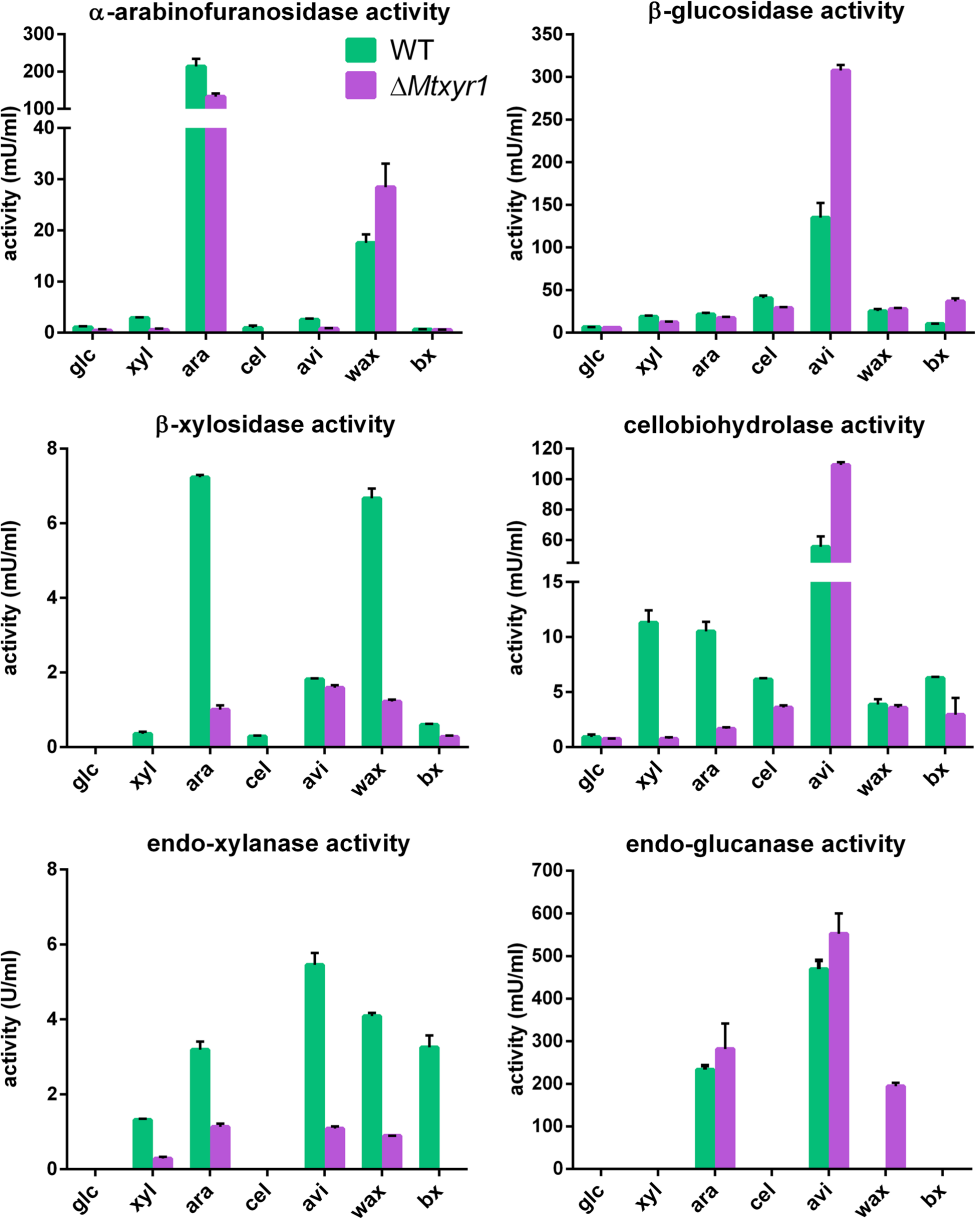
Endo- and exo-acting enzyme activities after 24 h of growth of the wild-type and Δ*xlr1* strain on lignocellulose related substrates. Strains were grown for 24 h on d-glucose (glc), d-xylose (xyl), l-arabinose (ara), cellobiose (cel), Avicel (avi), wheat arabinoxylan (wax) and birchwood xylan (bx). Total enzyme activities were measured in culture filtrate samples as described in “Materials and Methods”. One unit of activity is defined as 1 µmol pNP (or azo dye) released from the substrate per minute per mL culture filtrate

Deletion of *Mtxyr1* negatively affected β-xylosidase (BXL) and endo-xylanase (XLN) activities after 24 h of growth on d-xylose, l-arabinose, wheat arabinoxylan and birchwood xylan (Fig. 3). Interestingly, BXL and XLN activities were higher on l-arabinose than on d-xylose in the wild type after 24 h of growth. α-Arabinofuranosidase (ABF) activity was higher in the wild type on l-arabinose and wheat arabinoxylan compared to the other substrates. However, deletion of *Mtxyr1* resulted in only a 40% reduction of ABF activity on l-arabinose, while there was an increase in the ABF activity of the mutant of 50% on wheat arabinoxylan.

We observed no reduced activities of β-glucosidases (BGL) and endo-glucanases (EGL) in Δ*Mtxyr1* after 24 h of growth on all substrates (Fig. 3). EGL activity was only detected in 24 h samples of both the wild-type and Δ*Mtxyr1* grown on l-arabinose and Avicel and in Δ*Mtxyr1* on wheat arabinoxylan. Deletion of *Mtxyr1* resulted in a strong reduction in cellobiohydrolase (CBH) activity levels after the 24 h transfer on d-xylose and l-arabinose, but CBH activity was not or only weakly affected on cellobiose and Avicel. The cellulolytic enzyme activities are higher in the deletion strain on Avicel (1.5- to 2-fold for BGL and CBH).

### MtXyr1 is mainly involved in regulation of expression of xylanolytic genes on both **d-**xylose and **l-**arabinose

RNA-seq was used to study the effect of the *Mtxyr1* deletion on transcript levels of genes encoding Carbohydrate Active enZymes (CAZymes). The wild-type and Δ*Mtxyr1* strains were pre-grown in CM medium containing 2% d-fructose and aliquots of the mycelium were transferred for 2 h to minimal media containing 25 mM d-glucose, d-xylose or l-arabinose and for 2 and 8 h to minimal medium containing 1% wheat arabinoxylan. These time points were chosen to reflect the initial response (2 h) of the strains to the new conditions selected and how this response alters over time (8 h).

**Fig. 4:**
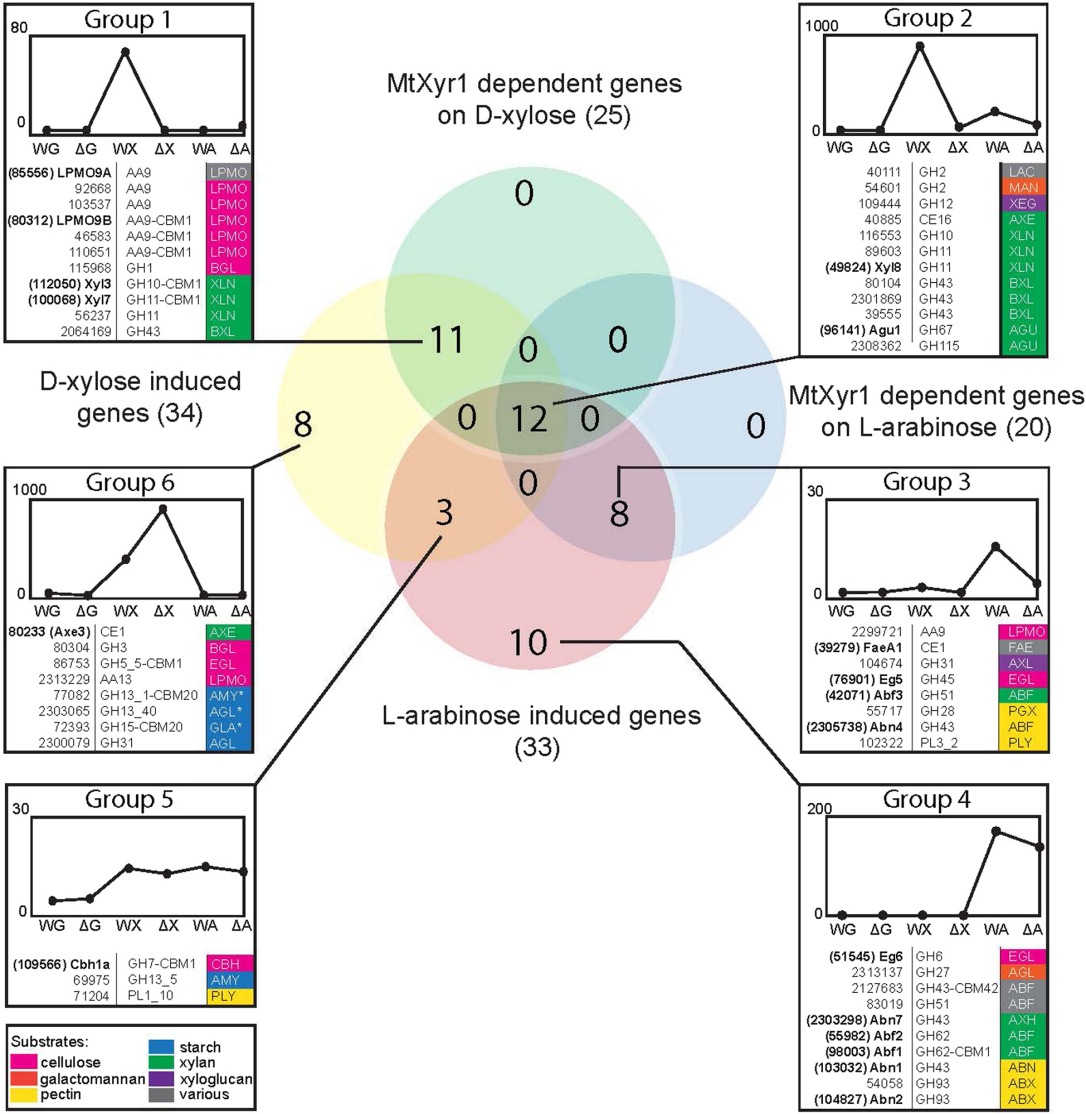
Venn diagram showing the JGI numbers, CAZy classifications and (determined or predicted) enzyme activities encoded by differentially expressed genes (DEGs) expected to be involved in plant cell wall degradation. The CAZy genes that are up-regulated (> twofold, *p* value > 0.01) in the 2 h d-xylose or l-arabinose samples compared to the 2 h d-glucose samples of the *M. thermophila* wild-type strain are designated ‘d-xylose induced’ or ‘l-arabinose induced’ genes. The MtXyr1 dependent genes are genes down-regulated (> twofold, *p* value > 0.01) in the Δ*Mtxyr1* strain on d-xylose or l-arabinose compared to the wild-type strain on d-xylose or l-arabinose. The genes containing an asterisk are genes that were up-regulated in the Δ*Mtxyr1* strain. The graphs show the gene expression patterns for the genes of each of the seven groups. DEGs were grouped and of each group the average RPKM per conditions was calculated and plotted in a graph (*y*-axis: RPKM value and *x*-axis: strain and substrate. W = wild-type strain, Δ = Δ*Mtxyr1* strain, *G* = 2 h d-glucose, *X* = 2 h d-xylose and *A* = 2 h l-arabinose). The enzyme activities were predicted based on manual annotation and phylogenetic analysis (see Additional file 2 for annotation results and enzyme abbreviations). In bold, the enzymes that have been characterized in the *M. thermophila* C1 strain. The color scheme (see legend) represents the specific plant polysaccharide the enzyme is predicted to act on in pink, orange, yellow, blue, green and purple. In gray (various), the enzymes that act on more than one specific plant polysaccharide. If there is no color assigned, the substrate of which the enzyme is active on remains unknown

First, we studied the transcript levels of the CAZy genes that contain at least one predicted auxiliary activity (AA), carbohydrate esterase (CE), glycoside hydrolase (GH) or polysaccharide lyase (PL) domain (Additional file 2). A selection was made of the CAZy families that are known or predicted to be involved in plant polysaccharide degradation in *Aspergillus nidulans* [32]. A cutoff fold change of the data from the mutant compared to the wild type of > 2.0 and a *p* value of ≤ 0.01 was used to identify differentially expressed genes (Fig. 4 and Additional file 2). Only genes expressed above 5 RPKM in at least one sample were included in the expression profile analysis in the next paragraphs.

Transcript levels of 34 genes were induced on d-xylose compared to d-glucose in the wild type, while 15 of these genes were also induced on l-arabinose (Fig. 4). Most genes induced on both pentoses are known/predicted to be involved in xylan degradation. The 20 CAZy genes that are only induced on d-xylose are known/predicted to be involved in xylan, cellulose and starch degradation (the genes of group 1 and 6, see Fig. 4).

Transcript levels of the CAZy genes of group 1, 2 and 3 were all significantly decreased in Δ*Mtxyr1* (Fig. 4).

As expected, this group consists of genes encoding hydrolytic enzymes (GH10, GH11, GH43) involved in xylan degradation, but remarkably also contains a large number of LPMO’s. The expression of 6 out of 24 LPMO genes was lower in Δ*Mtxyr1* on d-xylose (Fig. 4, group 1). Two of these group 1 AA9 genes, encoding *Mt*LPMO9A and *Mt*LPMO9B from *M. thermophila* C1, were studied before and were shown to be active toward regenerated amorphous cellulose (RAC) and cellulose, whereas *Mt*LPMO9A was also active toward xylan associated with RAC, xyloglucan and mixed glucan [33].

Group 2 contains genes of which transcript levels were decreased in Δ*Mtxyr1* on both d-xylose and l-arabinose (Fig. 4). Almost all genes encode main chain cleaving and accessory enzymes involved in xylan degradation, except for a predicted GH2 β-mannosidase and a predicted GH12 xyloglucan-specific endo-glucanase. Group 3 contains genes that had low RPKM values (between 5 and 30), but they were l-arabinose induced and transcript levels were significantly decreased in Δ*Mtxyr1* on l-arabinose. Most genes of this group are involved in xylan and pectin degradation, for example those encoding the α-L-arabinofuranosidases Abf3 and Abn4, and the feruloyl esterase FaeA1 [34, 35].

Genes that were not dependent on *Mt*Xyr1 are presented in group 4, 5 and 6 (Fig. 4). Group 4 consists of a set of genes only induced on l-arabinose of which the transcript levels were not influenced by *Mt*Xyr1. These genes mainly encode arabinan- and arabinoxylan-degrading enzymes. Group 5 includes three genes that encode a GH7 cellobiohydrolase (Cbh1a), a predicted GH13_3 α-amylase and a predicted PL1_10 pectate lyase which show increased transcript levels on both d-xylose and l-arabinose, but were not affected by deletion of *Mtxyr1*. Group 6 includes genes expressed in the presence of d-xylose that are not *Mt*Xyr1 dependent. Almost all those genes are involved in starch and cellulose degradation. Only one of these genes, encoding the CE1 acetylxylan esterase Axe3, is active toward acetylated xylans [36]. Three starch-degrading genes (a predicted GH13 α-amylase, a starch-binding GH15 glucoamylase and a GH13 α-glucosidase) from this group were significantly up-regulated in Δ*Mtxyr1* on d-xylose, likely as a response to the inability to utilize d-xylose.

In summary, transcript levels of none of the endo-glucanases or cellobiohydrolases belonging to the GH5, 6 and 7 families were affected by the deletion of *Mtxyr1* on d-xylose or l-arabinose at the 2 h time point (Fig. 4). The transcript level of a predicted GH5_5 endo-glucanase did increase on d-xylose, but was unaffected in the absence of *Mtxyr1* (Fig. 4, group 6). There was only one GH45 endo-glucanase (Eg5) that was l-arabinose induced and down-regulated in Δ*Mtxyr1* on l-arabinose (Fig. 4, group 3).

### Not all xylanolytic genes remain down-regulated in Δ*Mtxyr1* during growth on wheat arabinoxylan

To identify CAZy genes regulated by Xyr1 in *M. thermophila* on wheat arabinoxylan, we used RNA-seq to determine changes in mRNA levels in the wild type and Δ*Mtxyr1* at two time points, 2 h and 8 h. CAZy genes were considered to be time-independently regulated by *Mt*Xyr1, if at both time points the transcript levels were more than twofold decreased in Δ*Mtxyr1* compared to the wild type.

**Fig. 5:**
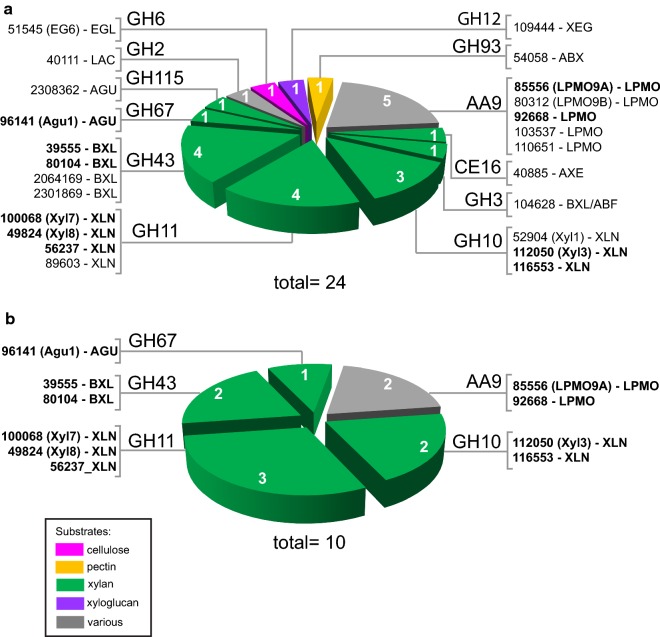
The numbers of down-regulated genes per CAZy family in the Δ*Mtxyr1* strain on wheat arabinoxylan compared to the wild-type strain. **a** Down-regulated (> twofold, *p* value > 0.01) CAZy genes at the 2 h time point and **b** 8 h time point in the Δ*Mtxyr1* strain. Only the GH, AA, CE or PL families predicted to be involved in plant cell wall degradation per gene are shown. For additional information (enzyme abbreviations and the presence of CBM modules, see Additional file 2). In gray (various/unknown), the enzymes that act on more than one specific plant polysaccharide or of which the substrate it acts on remains unknown

The expression of 24 CAZy genes was significantly lower in the Δ*Mtxyr1* strain at 2 h on wheat arabinoxylan (Fig. 5a). Many of these genes encode enzymes involved in the degradation of xylan, including seven known/predicted GH10 and GH11 endo-xylanases and nine predicted xylanolytic accessory enzymes. The decrease in transcript levels of a set of LPMO genes that was observed at the 2 h time point on d-xylose in Δ*Mtxyr1* (Fig. 4; group 1) was also observed on wheat arabinoxylan at the 2 h time point (Fig. 5a), except for one predicted AA9 LMPO (JGI: 46583). Initially, deletion of *Mtxyr1* had a negative effect on transcript levels of three other CAZy genes involved in the degradation of cellulose (GH6 endo-glucanase EG6) and of xyloglucan (JGI: 109444; a predicted GH12 xyloglucan endo-glucanase) as well as in pectin degradation (JGI: 54058; a GH93 exo-arabinanase). However, transcript levels did not remain low at the 8 h time point in the Δ*Mtxyr1* strain (Fig. 5b). Expression of only 10 of these 24 genes remained significantly lower in the Δ*Mtxyr1* strain at 8 h. These CAZy genes encode mainly xylan-degrading enzymes including five known/predicted GH10 and GH11 endo-xylanases (Xyl3, Xyl7, Xyl8, JGI: 56237 and JGI: 116553), two predicted GH43 β-xylosidases (JGI: 39555 and JGI: 80104), one α-glucuronidase (Agu1) and two LPMO’s (*Mt*LPMO9A and JGI: 92668).

**Fig. 6:**
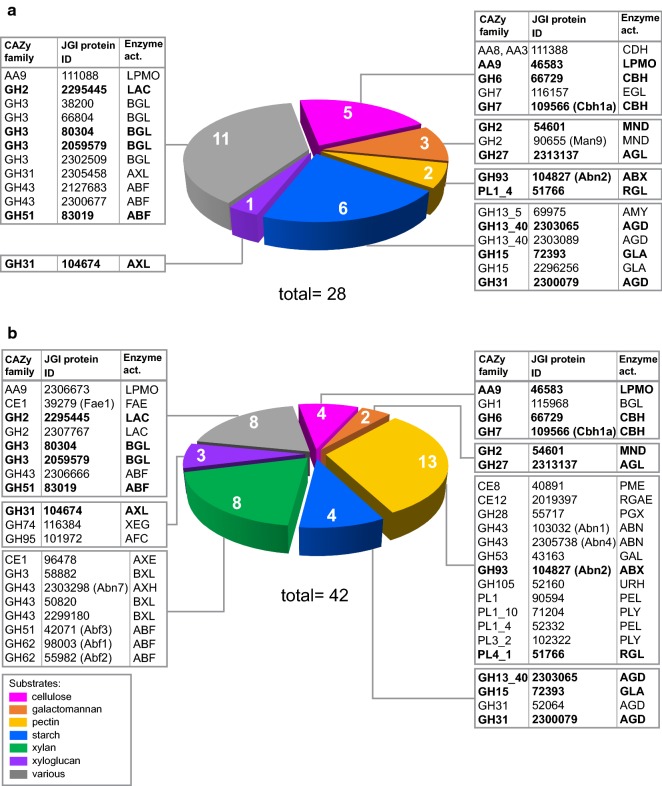
Up-regulated CAZy genes in the ΔMtxyr1 strain during 2 and 8 h of growth on wheat arabinoxylan compared to the wild-type strain. **a** Up-regulated (> twofold, *p* value > 0.01) CAZy genes at the 2 h time point and **b** 8 h time point in the ΔMtxyr1 strain. In bold, 15 CAZy genes that are up-regulated at both time points. Only the GH, AA, CE or PL families predicted to be involved in plant cell wall degradation per gene are shown. For additional information, i.e., predicted or known enzyme activity abbreviations (Enzyme act.) and the presence of CBM modules, see Additional file 2. In gray (various), the enzymes that act on more than one specific plant polysaccharide

Interestingly, transcript levels of many genes were significantly increased in Δ*Mtxyr1* compared to the wild type at both time points on wheat arabinoxylan (Fig. 6a). A total of 28 CAZy genes were up-regulated at 2 h. Among these CAZy genes, there is a set of genes predicted to be involved in starch degradation (six genes encoding two predicted GH13_40 α-glucosidases, a GH31 α-glucosidase, a GH13_5 amylase and two GH15 glucoamylases), a set of genes involved in cellulose degradation (five genes including a GH6 and a GH7 cellobiohydrolase, a GH7 endoglucanase, an AA9 LPMO and an AA8/AA3 cellobiose dehydrogenase) and a set involved in galactomannan degradation (three genes including two GH2 β-mannosidases and a GH27 α-galactosidase). The other 14 up-regulated CAZy genes in Δ*Mtxyr1* at 2 h were predicted to be mainly exo-acting enzymes acting on xyloglucan, pectin and various substrates.

At 8 h, the number of up-regulated CAZy genes increased to 42 (Fig. 6b), including sets of CAZy genes predicted to be involved in degradation of xylan (eight genes) and pectin (13 genes). Among these genes are those encoding enzymes releasing arabinan oligomers and l-arabinose. Some of these, *abn1*, *abn2*, *abf1* and *abf2*, were also up-regulated on l-arabinose, whereas transcript levels were unaffected by deletion of *Mtxyr1* (Fig. 4; group 4).

### Deletion of *Mtxyr1* also affects the transcription levels of some sugar transporter-encoding genes

**Fig. 7:**
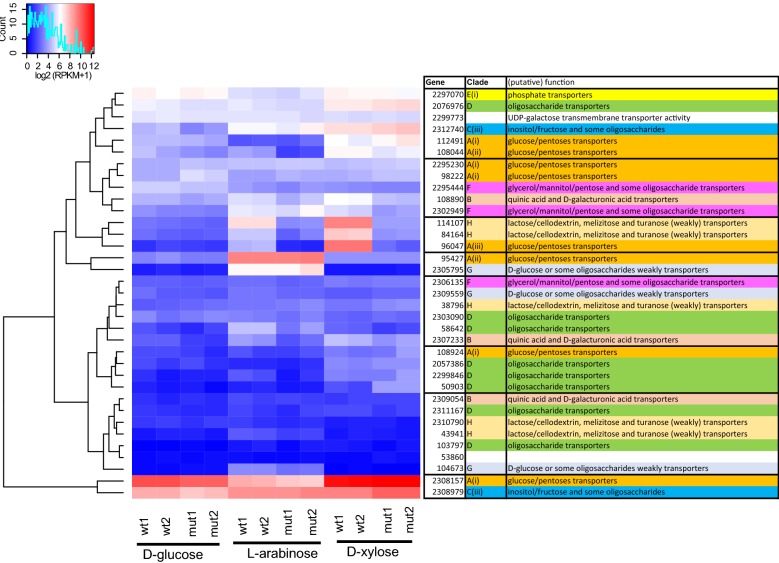
Hierarchical clustering of significantly differentially expressed putative sugar transporter genes in the Δ*Mtxyr1* strain. The wild-type (wt) and Δ*Mtxyr1* (mut) strain were grown for 2 h on d-glucose, l-arabinose and d-xylose. The color code represents the logged expression values (RPKM + 1) of both biological duplicates (named 2.1 and 2.2). The gene column provides the identifier (JGI protein numbers) of the published genome, the clade represents the sugar transporter subgroup according to de Vries et al. [41], while the third column contains a prediction of function of the putative *M. thermophila* transporter gene (see Additional file 4 for more information)

The effect of deleting the *Mtxyr1* gene on transcript levels of 45 putative transporter genes in the *M. thermophila* genome was also analyzed. When all conditions and both strains were compared, 35 differentially expressed genes were detected (Fig. 7). The comparison for the three monosaccharides used is depicted in Fig. 7. The transcript levels of five putative transporter genes were induced on d-xylose and significantly decreased in Δ*Mtxyr1* after 2 h on d-xylose (Fig. 7 and Additional file 1: Fig S3). These include two genes of the second cluster (JGI: 108890 and 2302949) and three genes of the third cluster (JGI: 84164, 114107 and JGI: 96047) (Fig. 7). The latter gene is an ortholog of the *A. nidulans* d-xylose transporter (XtrD) that has been shown to be XlnR dependent in the presence of d-xylose in this fungus [37]. The two cluster 2 genes showed a highly similar transcription profile: both genes were induced on d-xylose, down-regulated in Δ*Mtxyr1* on d-xylose and up-regulated in the deletion strain at both time points on wheat arabinoxylan. JGI: 108890 is an ortholog of the *A. niger* d-galacturonic acid transporter (GatA) [38], while JGI: 2302949 is an ortholog of the d-xylose-induced transporter gene AN4148 (XtrE) of *A. nidulans* [37]. Transcript levels of JGI: 96047 were highly induced on d-xylose (400-fold compared to d-glucose), also induced on l-arabinose and wheat arabinoxylan (Additional file 1: Fig. S3), but down-regulated in the Δ*Mtxyr1* strain in all conditions. The transcript levels of the other two cluster 3 genes, JGI: 84164 and JGI: 114107, were lower in Δ*Mtxyr1* on l-arabinose and d-xylose. JGI: 114107 is an ortholog of the *N. crassa* cellodextrin/xylodextrin transporter CDT-2 gene that has been shown to be regulated by XLR-1, CLR-1 and CLR-2 during growth of *N. crassa* on cellulose and xylan [30, 39, 40]. JGI: 84164 is predicted to belong to the clade H of sugar transporters, including lactose permeases, cellodextrin transporters and members that are able to transport melezitose and turanose [41].

There were 11 putative transporter genes induced on l-arabinose (Additional file 3). Transcript levels of the two genes belonging to cluster 4 (JGI: 95427: the *M. thermophila* l-arabinose transporter (*Mt*LAT-1) and JGI: 2305795) were highly induced (a 60- and 96-fold change, respectively, compared to d-glucose) and remained unchanged in Δ*Mtxyr1* on l-arabinose. Transcript levels of five l-arabinose-induced transporter genes (JGI: 58642, JGI: 84164, JGI: 96047; the *A. nidulans* XtrD ortholog, JGI: 114107; the *N. crassa* CDT-2 ortholog and JGI: 2307233) decreased in the Δ*Mtxyr1* strain on l-arabinose.

Cluster 2, 4 and 5 include sugar transporter genes of which transcript levels highly increased in Δ*Mtxyr1* at 8 h on wheat arabinoxylan (Additional file 1, Fig. S3) and for several genes at both 2 h and 8 h. Clusters 6 and 7 contain lowly expressed genes and only minor changes in transcript levels occurred.

### Deletion of *Mtxyr1* affects the transcription of genes involved in the pentose catabolic pathway (PCP) and pentose phosphate pathway (PPP)

**Fig. 8:**
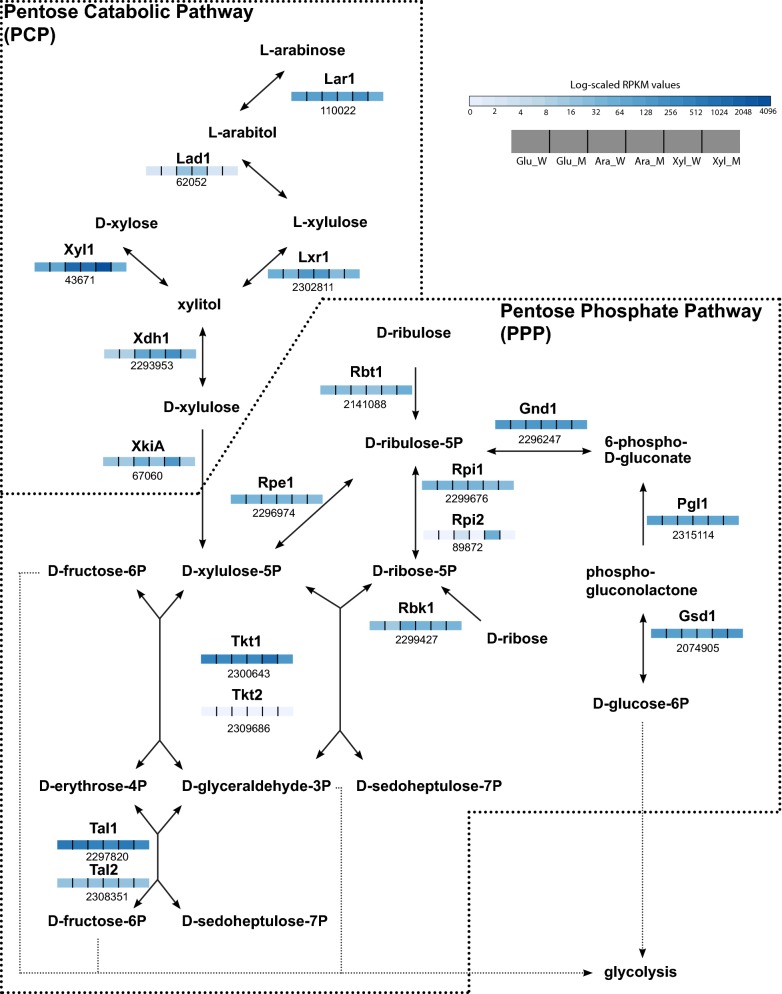
Schematic presentation of the expression of the genes from pentose catabolism. The figure represents a schematic view of the pentose catabolic pathway in which the (putative) *M. thermophila* genes involved are indicated next to the catalytic step carried out by their corresponding enzymes. The gene names are indicated above the boxes and the corresponding protein numbers from the published JGI genome of this species below the boxes. The boxes themselves reflect the expression of the genes on three substrates (d-glucose, l-arabinose and d-xylose) both in the wild type and the *Mtxlr1* mutant. Expression values are expressed as log-scaled RPKM values

Deletion of *Mtxyr1* significantly decreased the transcript levels of ten genes predicted to be involved in primary metabolism during growth of *M. thermophila* on d-xylose (Fig. 8, Additional files 1: Fig. S4 and 4). This included four genes predicted to encode the pentose catabolic pathway (PCP) enzymes: d-xylose reductase (Xyl1; JGI: 43671), xylitol dehydrogenase (Xdh1; JGI: 2293953), d-xylulose kinase (Xki1; JGI: 67060) and l-arabinose/d-galacturonic acid reductase (Lar1; JGI: 110022). Transcript levels of two pentose phosphate pathway (PPP) genes, encoding a putative transketolase (Tkt1; JGI: 2300643) and a ribose-5-phosphate isomerase (Rpi2; JGI: 89872), and four putative glycolytic genes (JGI: 39694, triose-phosphate isomerase; JGI: 84302, an alcohol dehydrogenase; JGI: 2082283, an aldose 1-epimerase and JGI: 2298452, a d-fructose-biphosphate aldolase) were also decreased in Δ*Mtxyr1* on d-xylose.

Reduced transcript levels of only five genes were observed in Δ*Mtxyr1* on l-arabinose: *xki1*, *rpi2* and the aldose 1-epimerase gen (JGI: 2082283) and d-fructose-biphosphate aldolase (JGI: 2298452)-encoding genes mentioned above and an aldehyde dehydrogenase gene (JGI: 2140820). The expression of five out of six PCP genes (with the exception of *lar1*) was induced in the wild type in the presence of l-arabinose or arabinoxylan compared to d-glucose (Additional file 1: Table S1).

Deletion of *Mtxyr1* also negatively affected the expression of genes predicted to be involved in the PCP and PPP during 2 and 8 h of growth on wheat arabinoxylan. Transcript levels of *xyl1* were down-regulated at both 2 h and 8 h, while expression of *xki1* (JGI: 67060) was only down-regulated at 2 h. The transcript levels of two PPP genes, *tkt1* (JGI: 2300643) and *rpi2* (JGI: 89872), were also decreased in Δ*Mtxyr1* on wheat arabinoxylan after 2 h.

Transcript levels of many genes were observed to be significantly increased in Δ*Mtxyr1* after 2 h and/or 8 h on wheat arabinoxylan, reflecting metabolism utilizing l-arabinose. These genes are predicted to be involved in central metabolism (i.e., glycolysis and the PPP), the metabolism of d-mannose, the d-galacturonic acid pathway, the L-rhamnose pathway and the Leloir pathway. Transcript levels of *lar1* (JGI: 110022), *rpi2* (JGI: 89872) and a triose-phosphate isomerase gene (JGI: 39694) were only increased after 8 h on wheat arabinoxylan in Δ*Mtxyr1*.

## Discussion

Fungi have developed complex mechanisms to adjust their carbon metabolism to minimize energy demands. In the presence of easily metabolized carbon sources, such as glucose, the expression of genes necessary for utilization of alternative carbon sources is repressed [42]. d**-**Xylose and **l-**arabinose are important components of the plant cell wall polysaccharides. Those pentoses are able to act as inducers of the expression of genes encoding plant biomass-degrading enzymes, through the regulation of the xylanolytic activator XlnR [26]. This activator (Xyr1 in *M. thermophila*) is not only involved in the regulation of CAZy-encoding genes and a set of sugar transporters, but also contributes to regulation of genes in the pentose catabolic pathway (PCP) and pentose phosphate pathway (PPP) [43–45]. Xyr1 homologs are commonly found in ascomycete fungi. However, induction and regulation of genes involved in xylan and cellulose degradation are significantly different among species [25, 30, 46]. No detailed analysis has been published to date about how this transcription factor regulates the xylanolytic system in the industrially important thermophilic fungal enzyme producer *M. thermophila*. In our present study, we aimed at obtaining a comprehensive understanding of how an *xyr1* deletion affects gene expression in this fungal host.

Growth phenotype analysis of the Δ*Mtxyr1* strain revealed severely reduced growth on d**-**xylose and birchwood xylan. Uptake experiments using transfer of fructose pre-grown mycelia showed that d-xylose uptake was impaired in the mutant strain but l-arabinose uptake was only marginally slowed down. This correlates well with the expression results which showed that four genes involved in the PCP (*xyl1*, *xdh1, xki1* and *lar1*) were significantly down-regulated, suggesting that those genes are under control of Xyr1. This is similar to what was observed in *T. reesei* where the deletion of Xyr1 showed a strong reduction in growth on d**-**xylose [47], whereas in that case the whole pathway turned out to be regulated by Xyr1 [29]. In the case of *M. thermophila lad1* and *lxr1*, the expression levels are not affected by the Xyr1 deletion. A reduced growth on d**-**xylose associated with a low expression of PCP genes, *xyr1, prd1, lad1* and *xdh1*, was also reported for the *M. oryzae* deletion mutant of Xlr1, although this regulator does not control the xylanolytic system [48]. On the other hand, for *Aspergillus* it has been demonstrated that the growth on d**-**xylose is only affected when the transcriptional regulators XlnR and AraR are both deleted [49], suggesting that the regulation of the PCP partially depends on XlnR.

An important response was also observed in the transcription of genes involved in the pentose phosphate pathway. Expression of the ribose 5-phosphate isomerase (*rpi2*)*-*encoding gene and a putative transketolase (*tkt1)* was significantly lower in the *ΔMtxyr1* mutant upon transfer from fructose to d**-**xylose, indicating direct regulation by Xyr1. As previously proposed [44], the transcription regulators XlnR and AraR also regulate some PPP genes in *A. niger*.

Growth of *ΔMtxyr1* on l-arabinose was slightly reduced and a lower expression of *xki1* and *rip2* was observed. This could be explained by the small amount of d-xylose present in commercial l**-**arabinose [50] and this possibly induced Xyr1-regulated genes in the wild type. There is no difference in *lad1* and *lxr1* expression between the reference strain and the *ΔMtxyr1* mutant representing two steps of the PCP specific for l-arabinose utilization. This is the case both in d-xylose and l-arabinose, indicating that Xyr1 is not involved in l-arabinose utilization.

Reduction in growth on arabinoxylan was also pronounced in *ΔMtxyr1*. The transcript level of *xkiA* was only down-regulated at the 2 h time point, while expression of *xyl1* decreased at 8 h. Since wheat arabinoxylan is a substrate mainly containing d-xylose and l-arabinose, it is likely that *M. thermophila* takes up d**-**xylose preferentially, indicating the expected profile of down-regulated Xyr1-dependent genes at early time points. We demonstrated (unpublished results) that when providing a xylose/arabinose mixture, xylose was preferentially taken up during the first 12 h of growth in the wild type. In the *Mtxyr1* deletion strain, d-xylose was hardly taken up but did gradually disappear from the medium in the presence of l-arabinose indicating that uptake of d-xylose requires energy which is provided by the co-substrate.

The question whether Xyr1-dependent transporters are still involved in the slow d**-**xylose uptake observed under these conditions can only be answered by systematically eliminating these transporters.

Xylanolytic activities present in the culture filtrate were significantly affected in *ΔMtxyr1* after 24 h of growth on d-xylose, l-arabinose, arabinoxylan and birchwood xylan. Previously, it was shown that overexpression of Xyr1 in *M. thermophila* ATCC 42464 mainly resulted in an increased xylanase activity both in glucose and in corncob-containing media. However, the filter paper activity and endoglucanase activities were not affected in corncob- and glucose-containing medium indicating that Xyr1 plays no or only a limited role in regulating cellulolytic gene expression [31].

To gain insight into the regulation of CAZy genes, the transcriptional responses during growth on d-glucose, d-xylose, l-arabinose and wheat arabinoxylan (2 and 8 h) were analyzed in the *ΔMtxyr1* mutant (Fig. 4). Our results demonstrated that Xyr1 is required for the expression of hemicellulolytic genes and a subset of mainly AA9 LPMO genes which are usually considered to play a role in cellulose oxidation. d**-**xylose activates the expression of these genes (Group 1 and 2).

The xylanolytic genes comprise endo- and exo-acting encoding activities and some auxiliary functions as expected. The difference in expression profile of the genes of Group 2 and Group 1 is most likely due to d**-**xylose impurities present in **l-**arabinose in combination with differences in carbon catabolite repression of the target genes. Interestingly, among the genes in Group 1, 6 AA9 LPMOs were found representing a substantial number of the 22 AA9 LPMO genes reported before to be present in the *M. thermophila* genome [10]. A recent study showed that the activity of one of the lytic polysaccharide monooxygenase (MtLPMO9A) from *M. thermophila* C1 cleaves β-(1 → 4)-xylosyl bonds in xylan, generating oxidized xylo-oligosaccharides, whereas it simultaneously cleaves β-(1 → 4)-glucosyl bonds in cellulose having a synergistic interaction with endoglucanase I [51]. As this specific LPMO is active on xylan, our transcriptomic data suggests that other LPMOs responding to Xyr1 might also be involved in oxidative cleavage of xylan. Additionally, one GH1 β-glucosidase gene (Group 1), an endo-xyloglucanase and a mannosidase (Group 2) were down-regulated in the presence of d-xylose, showing that at least a few cellulolytic genes are regulated by Xyr1. Similarly, in *N. crassa*, Xlr-1 is absolutely required for the expression of hemicellulose genes and those involved in d**-**xylose metabolism, while cellulase genes are slightly affected [52]. It has been reported that two transcription factors CLR-1 and CLR-2 are the predominant regulators of the expression of cellulase-encoding genes in *N. crassa* [24] as is probably the case in *M. thermophila*. Recently, a new specific regulator MHR1 was identified in *M. thermophila* ATCC 42464 and the results showed that an *Mtmhr1* deletion increased expression of the main cellulase genes (*cbh1*, *cbh2*, *egl3*) and also of *xyr1*. It is not yet understood whether this regulator has any connection with CRE1 and ACE1 [53].

Expression of two L-arabinofuranosidase genes (*abf3*, *abn4*) was significantly down-regulated in *ΔMtxyr1* on **l-**arabinose (Group 3). This indicates that Xyr1 also regulates a few genes of the arabinanolytic system. However, the expression of *abf1, abf2* and other arabinanolytic genes is not under control of Xyr1 (Group 4), indicating the requirement of another transcriptional regulator. In *Aspergillus*, the arabinanolytic regulator AraR controls the expression of genes encoding α-arabinofuranosidases (AbfA, AbfB) [54] Our results also show a group of genes involved in cellulose and starch degradation (Group 6) that were significantly up-regulated on d**-**xylose and are not MtXyr1 dependent.

During the early 2 h growth phase on wheat arabinoxylan, the majority of xylanolytic genes were down-regulated and a set of genes involved in starch, cellulose and pectin degradation were up-regulated in the Δ*Mtxyr1* strain. This might be caused by a compensation effect by other transcription factors that act in a coordinated manner to induce enzymes which would be able to recruit nutrients from other polymers over time.

## Conclusions

Although in the industrial fungi *A. niger, A. oryzae* and *T. reesei*, the Xyr1 homolog is involved in regulating the expression of the xylanolytic system and at least part of the cellulolytic system, this is not the case in *M. thermophila*. Xyr1 seems to be predominantly restricted to the xylanolytic system, as confirmed by the extracellular enzyme profiles determined 24 h after the transfer to the different carbon sources and by the significantly reduced XLN and BXL activities in the *Mtxyr1* deletion mutant. The effects of the Xyr1 deletion at the transcriptome level of the CAZy genes identifies lack or reduced expression of all core activities involved in hydrolyzing xylan.

ABF activity clearly depends on the presence of **l-**arabinose or on arabinoxylan and expression of the corresponding genes does not require Xyr1, but likely depends on the homolog of the Ara1 activator present in *T. reesei* and *M. oryzae.*

The transcriptome analysis upon transfer from fructose to the polysaccharide arabinoxylan carried out after 2 h and 8 h illustrates the dynamics of the adaptation process. At the 2 h time point, there is a considerable overlap both in number and identity between the Xyr1-dependent genes on **d-**xylose [35] and on arabinoxylan [24] compared with wild-type and the *Mtxyr1* deletion strain. The number of strictly Xyr1-dependent genes reduces to ten at the 8 h time point on arabinoxylan.

## Methods

### Strains, media and growth conditions

All *Myceliophthora thermophila* strains described in this study were grown on malt extract agar (MEA) plates at 37 °C for 7 days to obtain conidia. For growth phenotypic profiling, the WT and Δ*Mtxyr1* strain were grown on minimal medium agar plates (pH set to 6.0) containing AspA + N (70 mM NaNO_3_, 7 mM KCl, 11 mM KH_2_PO_4_), 2 mM MgSO_4_, trace elements (174 µM EDTA, 76 µM ZnSO_4_·7H_2_O, 178 µM H_3_BO_3_, 25 µM MnSO_4_·H_2_O, 18 µM FeSO_4_·7H_2_O, 7.1 µM CoCl_2_·6H_2_O, 6.4 µM CuSO_4_·5H_2_O and 6.2 µM Na_2_MoO_4_·2H_2_O), 1.6% agar–agar (Merck Company, Darmstadt, Germany), 10 mM of uridine (if necessary) and 25 mM of d-glucose, d-xylose, l-arabinose or cellobiose or 1% of birchwood xylan, wheat arabinoxylan, Avicel or cellulose. All sugar substrates were purchased from Sigma-Aldrich (St. Louis, MO, USA), except for cellobiose (Acros Organics, Belgium), wheat arabinoxylan (Megazyme, Wicklow, Ireland) and Avicel (Fluka Chemicals, Buchs, Switzerland). Strains were inoculated with 2 µL containing 500 spores and grown for 5 days at 37 °C.

In the sugar consumption experiments, the WT and Δ*Mtxyr1* strains were pre-grown in complete medium (pH 6.0) containing AspA + NH_4_ (35 mM (NH_4_)_2_SO_4_, 7 mM NaCl, 55 mM KH_2_PO_4_), 2 mM MgSO_4_, trace elements (as described above), 0.1% casamino acids, 4 μg/L biotin and 2% fructose buffered with extra addition of 70 mM KH_2_PO_4_. The strains were inoculated with 1·10^5^ spores per mL and the liquid cultures were incubated at 37 °C at 200 rpm in a rotary shaker. After 36 h of incubation, the mycelium was then transferred into shake flasks with minimal medium by filtering the pre-culture medium through Miracloth. The mycelium was quickly washed with minimal medium without sugar and approximately 1.0 g (wet weight) of mycelium was transferred to 50 mL liquid shake flask cultures. Minimal medium (pH 6.0) contained AspA + NH_4_, 2 mM MgSO_4_, trace elements, 0.1% casamino acids, 4 μg/L biotin and was buffered with 70 mM KH_2_PO_4_. In addition, 1% d-xylose or l-arabinose or a mixture of the both sugars (0.5% of each sugar) was added to the minimal medium. The liquid shake flasks were incubated at 37 °C at 200 rpm for 48 h and 2 mL medium samples were collected after 4, 8, 12, 24, 33 and 48 h.

For the extracellular activity measurements, the WT and Δ*Mtxyr1* strains were pre-grown in complete medium (pH 6.0) containing AspA + N, 2 mM MgSO_4_, trace elements, 0.1% casamino acids, 5 g/L yeast extract, 4 μg/L biotin and 1% d-glucose. Strains were inoculated with 1·10^5^ spores per mL and incubated at 37 °C at 200 rpm in a rotary shaker. After 36 h of incubation, pre-cultures were filtered using Miracloth, the mycelium was washed with the respective complete medium (without carbon source) and aliquots of wet mycelium were transferred to liquid shake flasks with minimal medium and as a carbon source 1% of d-glucose, d-xylose, l-arabinose, cellobiose, Avicel, wheat arabinoxylan or birchwood xylan, respectively, as described above and sampled after 24 h. Minimal medium (pH 6.0) containing AspA + NH_4_, 2 mM MgSO_4_, trace elements, 0.1% casamino acids and 4 μg/L biotin was buffered by an extra addition of 70 mM KH_2_PO_4_.

For RNA-seq analysis, the WT and Δ*Mtxyr1* strain were pre-grown in complete medium (pH 6.0) containing AspA + NH_4_, 2 mM MgSO_4_, trace elements, 0.1% casamino acids, 4 μg/L biotin and 2% d-fructose. Strains were inoculated with 1·10^6^ spores per mL and incubated at 37 °C at 200 rpm in a rotary shaker. After 36 h of incubation, aliquots of the mycelium were transferred to shake flasks with minimal medium and as a carbon source 25 mM of d-glucose, d-xylose or l-arabinose or 1% wheat arabinoxylan, respectively. Minimal medium (pH 6.0) contained AspA + NH_4_, 2 mM MgSO_4_, trace elements, 0.1% casamino acids and 4 μg/L biotin and was buffered by an extra addition of 70 mM KH_2_PO_4_. Mycelial samples were harvested after 2 h of incubation in all monosaccharide cultures and after 2 and 8 h of incubation in case of the wheat arabinoxylan culture. The mycelial samples were dried between tissue paper and directly frozen in liquid nitrogen for RNA extraction.

### Molecular biology methods

The deletion cassette used to create the *M. thermophila* Δ*xyr1* strain was constructed by fusion PCR. To delete *Mtxyr1* gene, the ORF was replaced by the coding sequence of the *M. thermophila pyr5* gene in the uridine C1 auxotrophic host strain (*pyr5*^−^). In the first PCR reactions, the upstream (fragment 1; F1) and downstream (fragment 2; F2) flanking genomic region of the *xlr1* gene, and the *pyr5* gene (fragment 3; F3) were amplified from C1 WT genomic DNA (Additional file 1: Fig. S1).

PCRs were performed using GoTaq^®^ Long PCR Master Mix (Promega, Madison, WI). To obtain the three fragments, all PCRs were prepared by using 12.5 μL of GoTaq Master Mix, 0.24 μM primer concentrations, 5 µg of template DNA (genomic *M. thermophila* DNA) and nuclease-free water to adjust the volume to 25 μL. The PCR conditions were as follows: DNA polymerase activation at 95 °C for 2 min was followed by 35 cycles of DNA melting at 94 °C for 30 s, annealing at 60 °C (F1 and F2 primer pairs) or 64 °C (F3 primer pair) for 30 s, extension at 72 °C at 1 kb per 60 s and with a final extension at 72 °C for 5 min. The flanking fragment F1 was amplified using the primer pair *xyr1*–5′_F (CTACTCCACGACTCCGTACTTGG) and *xyr1*–5′_R (ggacagcaccctgcgcgttgcctgccatggTGGAGATGGGGTCGTCAAGACC), containing the overlapping region with the *pyr5* gene in lowercase letters. The flanking fragment F2 was amplified using the primer pair *xyr1*–3′_F (gaagcgaagcaaagtctccgtcgacCTCCTCGTGTGTAACCGTGGG), containing the overlapping region with the *pyr5* gene in lowercase letters, and *xyr1*–3′_R (GCCACGACTTTGAGAAGCTCCC). The *pyr5* fragment F3 was amplified using the primer pair pyr5_F (CCATGGCAGGCAACGCGCAG) and pyr5_R (GTCGACGGAGACTTTGCTTCGCTTC). The fusion PCR was prepared by using 12.5 μL of GoTaq Master Mix, 0.24 μM primer concentrations (primer pair *xyr1*–5′_F and *xyr1*–3′_R), the three unpurified PCR products in a mixture at equal concentrations (1:1:1 molar ratio) and nuclease-free water to adjust the volume to 25 μL. The PCR conditions were as follows: 95 °C for 2 min was followed by 35 cycles of DNA melting at 94 °C for 30 s, annealing at 65 °C or 30 s, extension at 72 °C at 1 kb per 60 s and with a final extension at 72 °C for 5 min. The fusion PCR product (*Mtxyr1* deletion cassette) was purified using the Wizard^®^ SV Gel and PCR Clean-Up System (Promega, Madison, WI) and ligated into the pJET1.2/blunt cloning vector using the CloneJET PCR Cloning Kit (Thermo Fisher Scientific, Waltham, MA, USA) according to the manufacturer’s sticky-end protocol.

After co-transformation of the *M. thermophila* C1 *pyr5*^−^ strain with the *Mtxyr1* deletion cassette (see next paragraph), deletion of the *xlr1* gene was checked by PCR screening (Additional file 1: Fig. S1). Genomic DNA was obtained from three independent single deletion transformants (Δ*Mtxyr1*.*1*, Δ*Mtxyr1*.2 and Δ*Mtxyr1.3*). The PCR screen was performed using GoTaq^®^ Flexi DNA Polymerase (Promega, Madison, WI). The PCRs were prepared by using 5 μL of GoTaq^®^ Flexi buffer, 1.0 μM MgCl_2_, 0.20 μM of primer concentrations, 0.2 mM each dNTP, 20 ng of template DNA (genomic *M. thermophila* DNA), 0.125 μL of GoTaq^®^ Flexi polymerase and nuclease-free water to adjust the volume to 25 μL. The PCR conditions were as follows: 95 °C for 2 min was followed by 30 cycles of DNA melting at 94 °C for 30 s, annealing at 62 °C for 30 s, extension at 72 °C at 1 kb per 60 s and with a final extension at 72 °C for 5 min. The presence of the ORF of xlr1 was checked using the primers pair ORF_F (CAGGAAAGACCTTGCACAGC) and ORF_R (CACGGAAGTTACCGTTCTGG). The 5′ region of the *xlr1* deletion cassette was amplified using the primer pair DC5_F (TCAGGACAGCGTACAAGTAGTGG) and DCpyr5_R (GTGCTCCCTATCTGACAACTCC). The 3′ region was amplified using the primer pair DCpyr5_F (GCACAGCATCTCTTGAAGCG) and DC3_R (CTGCTTGATGCTCGATATGC).

One of gene deletion mutants (Δ*Mtxyr1.1* strain) was complemented with the wild-type gene copy, and a complementation vector was constructed containing the ORF of the *xyr1* gene with a 2000 bp promoter region and 1500 bp terminator region. The amplified PCR product was ligated into the pJET1.2/blunt cloning vector using the CloneJET PCR Cloning Kit (Thermo Fisher Scientific, Waltham, MA, USA) according to the manufacturer’s instructions.

### *Myceliophthora thermophila* transformation

About 10^6^ spores per mL were inoculated into 250 mL of complete medium (pH 6.0) containing AspA + NH_4_ (35 mM (NH_4_)_2_SO_4_, 7 mM NaCl, 55 mM KH_2_PO_4_), 2 mM MgSO_4_, trace elements (as described above), 0.1% casamino acids, 4 μg/L biotin and 2% fructose buffered with extra addition of 70 mM KH_2_PO_4_ and cultured at 37 °C at 200 rpm for 24 h. Young mycelia were harvested by vacuum filtration and washed with 100 mL NaCl/CaCl_2_ (0.6 M NaCl, 0.27 M CaCl_2_ 2H_2_O). About 2 g of mycelium was then dissolved in a protoplasting enzyme mix (20 mg Lysing Enzymes *Trichoderma harzianum,* 15 mg Driselase from Basidiomycetes sp. per gram of mycelium dissolved in 5 mL of NaCl/CaCl_2_) and incubated in a rotary shaker at 70 rpm at 30 °C. When enough protoplasts were formed, the mixture was filtered over a sterile Miracloth and washed with 22.5 mL NaCl/CaCl_2_. Next, 22.5 mL of cold STC (1.2 M sorbitol, 50 mM CaCl_2_. H_2_O, 35 mM NaCl, 10 mM Tris/HCl) was added to the suspension and mixed by gently inverting the tube a couple of times. The protoplasts were collected by centrifugation (10 min, 2500 rpm, 4 °C), resuspended in 50 mL of STC and centrifuged again. The supernatant was discarded and the pellet was carefully resuspended in the remaining STC (approximately 2 mL). For transformation, 100 μL of protoplast suspension, 1 μL of 0.5 M ATA (aurintricarboxylic acid ammonium salt) was mixed with 5 μg of deletion cassette DNA and incubated for 25 min. With a 10 mL pipette 8, 8 and 18 drops of 60% PEG4000 (60% PEG4000, 50 mM CaCl_2_ 2H_2_O, 35 mM NaCl, 10 mM Tris/HCl, 1 M Tris/HCl, pH 7.5) were added to the suspension and mixed gently by slowly inverting the tube between every step. After 20 min of incubation at room temperature, 50 mL of STC was added and centrifuged (10 min, 2500 rpm, 4 °C). The supernatant was removed and resuspended in the STC left over. Aliquots (0.1–0.3 mL) of transformed protoplast suspension were spread over selection plates containing 10 mM of uridine. Growing colonies were observed after 3 days at 37 °C.

### Sugar consumption and enzyme activity analysis

HPLC was used to determine the sugar concentrations in the medium samples collected after 4, 8, 12, 24, 33 and 48 h. Sugar analysis was performed as described previously on a Thermo Scientific 5000 + HPLC-PAD system [55]. The data obtained are the results of two independent biological replicates and for each replicate three technical replicates were assayed. Endo- and exo-acting enzyme activities in culture filtrates of transferred mycelia of the *M. thermophila* WT and Δ*Mtxyr1* strain to liquid shake flask cultures with lignocellulose-related substrates were measured using *p*NP- (*p*-nitrophenol) linked substrates (Sigma-Aldrich, St. Louis, MO, USA) to measure α-arabinofuranosidase, β-xylosidase, β-glucosidase and cellobiohydrolase activity. Azo-CM-cellulose and Azo-wheat arabinoxylan (Megazyme, Wicklow, Ireland) were used to measure endo-glucanase or endo-xylanase activity, respectively. Exo-acting enzyme activities were determined by measuring the amount of *p*NP released after 30–60 min by the culture filtrate with the *p*NP-linked substrates and standardized against a known concentration of *p*NP. Activities are expressed in mU/mL. The reactions contained between 5 µL and 30 µL of culture filtrate, 10 µL 0.1% pNP-linked substrate and 25 mM sodium acetate buffer, pH 5.0, in a total volume of 100 µL. All reactions were pipetted and mixed in a 96-wells microtiter plate, incubated at 50 °C and then stopped by the addition of 100 µL 0.25 M sodium carbonate. Absorbance was measured at 405 nm in a microtiter plate reader (FLUOstar OPTIMA, BMG Labtech). Endo-acting enzyme activities were measured in 100 µL reactions as described previously [56]. Absorbance was measured at 590 nm. Activities are expressed as mU/mL. All the enzyme activity data obtained are the results of two independent biological replicates and two technical replicates.

### RNA extraction and gene expression analysis

Total RNA was extracted from mycelium ground in a Tissue Lyser II (Qiagen, Venlo, the Netherlands) using TRIzol reagent (Invitrogen, Breda, the Netherlands) and purified with NucleoSpin^®^ RNA II Clean-up kit (Macherey–Nagel) with rDNase treatment. The RNA quantity and quality were checked with an RNA6000 Nano Assay using the Agilent 2100 Bioanalyzer (Agilent Technologies, Santa Clara, CA, USA). RNA samples were single-end sequenced at BGI Tech Solutions Co., Ltd. (Hong Kong, China) using the Illumina HiSeqTM 2000 platform (http://illumina.com). Raw reads were produced from the original image data by base calling. On average, 49 bp sequenced reads were constituted, producing 596 MB raw yields for each sample. After data filtering, the adaptor sequences, reads with unknown bases (*N*) > 10% and low-quality reads (more than 50% of the bases with quality value < 5%) were removed. Clean reads were mapped to the genome sequence of *M. thermophila v2.0* [17] using BWA/Bowtie [57, 58] with no more than two mismatches allowed in the alignment. On average, 90% of the clean reads were mapped to the genome. The gene expression level as reads per kilobase of exon per million fragments mapped (RPKM) was calculated by using the RSEM tool [59]. Differential expression was identified by CyberT Bayesian ANOVA algorithm [60]. A fold change of > 2 and *p* value of < 0.01 were used to identify differentially expressed genes. The heat maps were made by the “gplots” package of R software, with the default parameters: “Complete-linkage clustering method and Euclidean distance”.

The CAZymes and sugar transporter genes were extracted from the *M. thermophila* genome available online at the JGI MycoCosm database, https://genome.jgi.doe.gov/Spoth2 [61]. The carbon metabolism genes were identified by orthologous mapping with the well annotated genes in the *Aspergillus* Genome Database (http://www.aspergillusgenome.org/ [62] using a bidirectional best hit (BBH) method [63, 64].

## Supplementary information

**Additional file 1.** Combines Table S1 and Figure S1-S4.

**Additional file 2.** Differentially expressed CAZy genes involved in plant cell wall degradation.

**Additional file 3.** Differentially expressed genes involved in sugar transport.

**Additional file 4.** Differentially expressed genes involved in primary metabolism.

## Data Availability

The datasets generated and analyzed during the current study were deposited at the Gene Expression Omnibus (GEO) database [65] with Accession Number: GSE137286.
