# Supplementary material for: Myceliophthora thermophila Xyr1 is predominantly involved in xylan degradation and xylose catabolism
Source: Biotechnol Biofuels. 2019 Sep 16;12:220. doi: 10.1186/s13068-019-1556-y (PMC6745793; doi:10.1186/s13068-019-1556-y)
Supplement: Supplementary file 1 — Additional file 1. Combines Table S1 and Figure S1-S4. [file 13068_2019_1556_MOESM1_ESM.pdf]

Additional file 1.

Combines Table S1 and Figure S1-S4

Table S1. Putative metabolic genes from *Myceliophthora thermophila* based on homology to genes from *A. niger*. Induction of the *M. thermophila* genes is indicated in the last column (see Additional file 4 for more information).

Protein	Homolog in *A. niger*:	Putative function in *M. thermophila*	Transcript levels are induced on:
**Pentose Catabolic Pathway (PCP)**			
62052	LadA (An01g10920)	L-arabitol dehydrogenase	L-arabinose
2302811	LxrA (An08g01930)	L-xylulose reductase	L-arabinose
2293953	XdhA (An12g00030)	Xylitol dehydrogenase	L-arabinose, D-xylose, arabinoxylan
67060	XkiA (An07g03140)	Xylulose kinase	L-arabinose, D-xylose, arabinoxylan
43671	XyrA (An01g03740)	D-xylose reductase	L-arabinose, D-xylose, arabinoxylan
**D-galacturonic acid and L-rhamnose pathway**			
87557	GaaC (An02g07720)	2-keto-3-deoxy-L_galactonate aldolase	L-arabinose
103080	LraB (An13g00940)	L-KDR aldolase	arabinoxylan
**Pentose Phosphate Pathway (PPP)**			
89872	RpiB (An02g02930)	Ribose 5-phosphate isomerase	L-arabinose, D-xylose, arabinoxylan
2300643	TktA (An08g06570)	Transketolase	arabinoxylan
**Glycolysis**			
112014	An14g02160	Alcohol dehydrogenase	L-arabinose
57502	An02g09090	Aldose-1-epimerase (or mutarotase)	L-arabinose
55576	AdhA (An17g01530)	Alcohol dehydrogenase	L-arabinose
2315623	AcuF (An11g02550)	Phosphoenolpyruvate carboxykinase	L-arabinose, D-xylose, arabinoxylan
39694	An02g02920	Triose-phosphate isomerase	D-xylose
84302	An16g02510	Alcohol dehydrogenase	D-xylose
2082283	An11g10890	Aldose 1-epimerase (or mutarotase)	D-xylose, arabinoxylan
2298452	An14g04410	Fructose-bisphosphate aldolase	D-xylose, arabinoxylan
**TCA cycle**			
2309210	Lpd1 (An07g06840)	Dihydrolipoamide dehydrogenase	L-arabinose, arabinoxylan
2295514	IdpA (An18g06760)	Isocitrate dehydrogenase	L-arabinose, arabinoxylan
**Ethanol utilization pathway**			
2140820	AldA (An08g07290)	Aldehyde dehydrogenase	L-arabinose, arabinoxylan

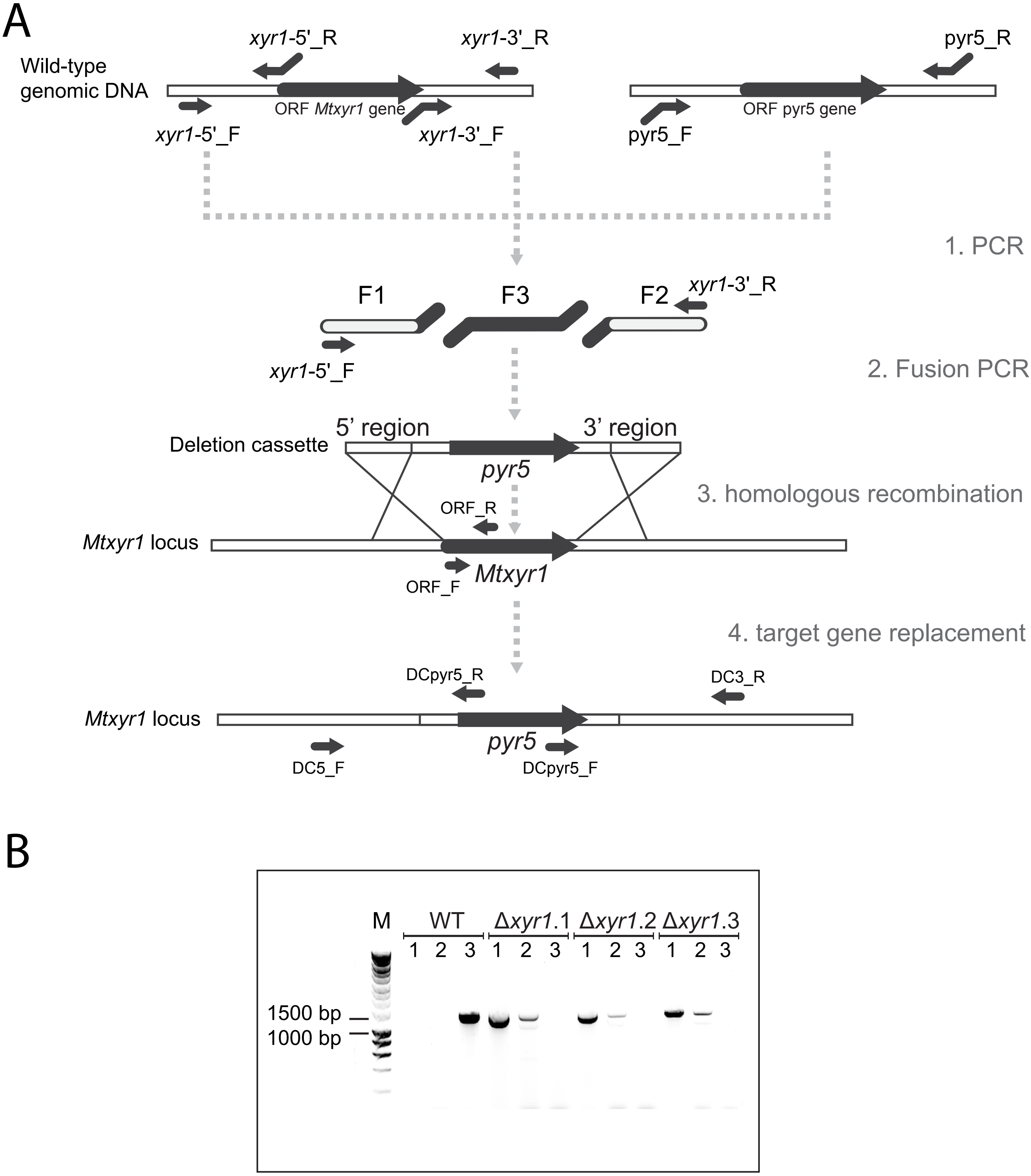

Figure S1. Deletion of the *Mtxyr1* gene in the *M. thermophila* C1 strain. A) Graphic representation of the deletion strategy (see the Materials and Methods section for the primer sequences. B) PCR screening of the wild-type (WT) strain and three independent transformants (Δ*xlr1.1*-3) that were obtained after transformation of *M. thermophila* WT with the *xlr1* deletion cassette. The lanes marked with 1: amplification of the 5' flanking region with primers DC5_F and DCpyr5_R. Lanes marked with 2: amplification of the 3' flanking region with primers DCpyr5_F and DC3_R. Lanes marked with 3: amplification inside the ORF of the Mt*xlr1* gene with primers ORF_F and ORF_R.

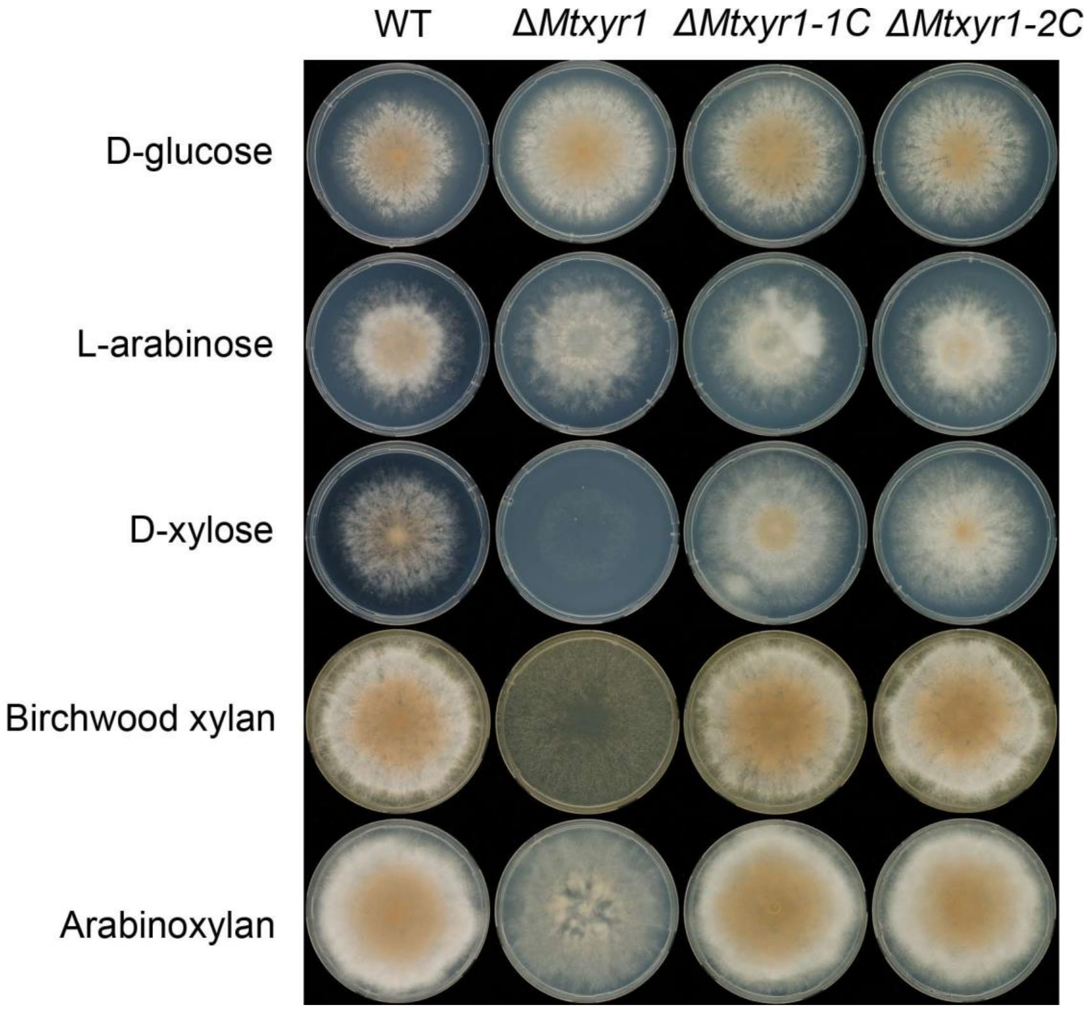

Figure S2. Growth phenotype of the *M. thermophila* wild type, Δ*xyr1* and complemented strains on agar plates. The wild-type strain, *Mtxyr1* deletion mutant (Δ*Mtxyr1*) and complemented strains (Δ*Mtxyr1*-1C and Δ*Mtxyr1*-2C; two independent transformants) were grown for 5 days at 37°C on solid media containing minimal medium with D-glucose, L-arabinose, D-xylose, birchwood xylan or wheat arabinoxylan.

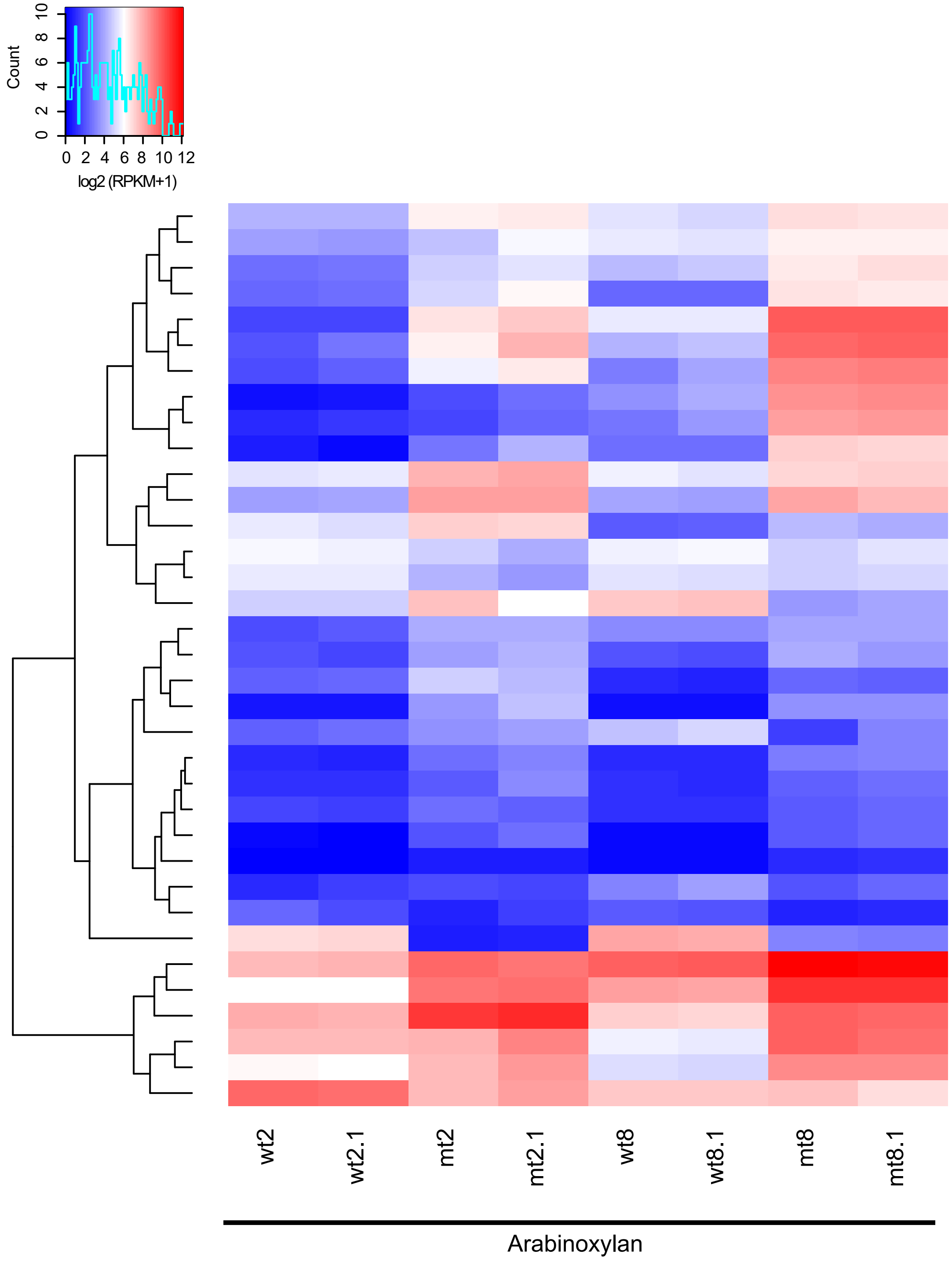

Gene	Clade	(putative) function
2295230	A(i)	glucose/pentoses transporters
98222	A(i)	glucose/pentoses transporters
2307233	B	quinic acid and D-galacturonic acid transporters
38796	H	lactose/cellodextrin, melizitose and turanose (weakly) transporters
2305795	G	D-glucose or some oligosaccharides weakly transporters
108044	A(ii)	glucose/pentoses transporters
58642	D	oligosaccharide transporters
2299846	D	oligosaccharide transporters
43941	H	lactose/cellodextrin, melizitose and turanose (weakly) transporters
104673	G	D-glucose or some oligosaccharides weakly transporters
2076976	D	oligosaccharide transporters
2302949	F	glycerol/mannitol/pentose and some oligosaccharide transporters
2303090	D	oligosaccharide transporters
2297070	E(i)	phosphate transporters
2299773		UDP-galactose transmembrane transporter activity
2312740	C(iii)	inositol/fructose and some oligosaccharides
2306135	F	glycerol/mannitol/pentose and some oligosaccharide transporters
2309559	G	D-glucose or some oligosaccharides weakly transporters
2295444	F	glycerol/mannitol/pentose and some oligosaccharide transporters
50903	D	oligosaccharide transporters
112491	A(i)	glucose/pentoses transporters
2310790	H	lactose/cellodextrin, melizitose and turanose (weakly) transporters
2311167	D	oligosaccharide transporters
2309054	B	quinic acid and D-galacturonic acid transporters
103797	D	oligosaccharide transporters
53860		
108924	A(i)	glucose/pentoses transporters
2057386	D	oligosaccharide transporters
96047	A(iii)	glucose/pentoses transporters
2308157	A(i)	glucose/pentoses transporters
95427	A(ii)	glucose/pentoses transporters
2308979	C(iii)	inositol/fructose and some oligosaccharides
84164	H	lactose/cellodextrin, melizitose and turanose (weakly) transporters
108890	B	quinic acid and D-galacturonic acid transporters
114107	H	lactose/cellodextrin, melizitose and turanose (weakly) transporters

Figure S3. Hierarchical clustering of significantly differentially expressed putative sugar transporter genes in the Δ*Mtxyr1* strain. The wild type and Δ*Mtxyr1* strain were grown for 2 and 8 h on wheat arabinoxylan. The color code represents the logged expression values (RPKM+1) of both biological duplicates (named 2 and 2.1 or 8 and 8.1). Behind the JGI protein numbers, the corresponding transporter clade according to de Vries et al., 2017 [54] is given, while the third column contains a prediction of function of the putative M. *thermophila* transporter gene (see Additional file 3 for more information).

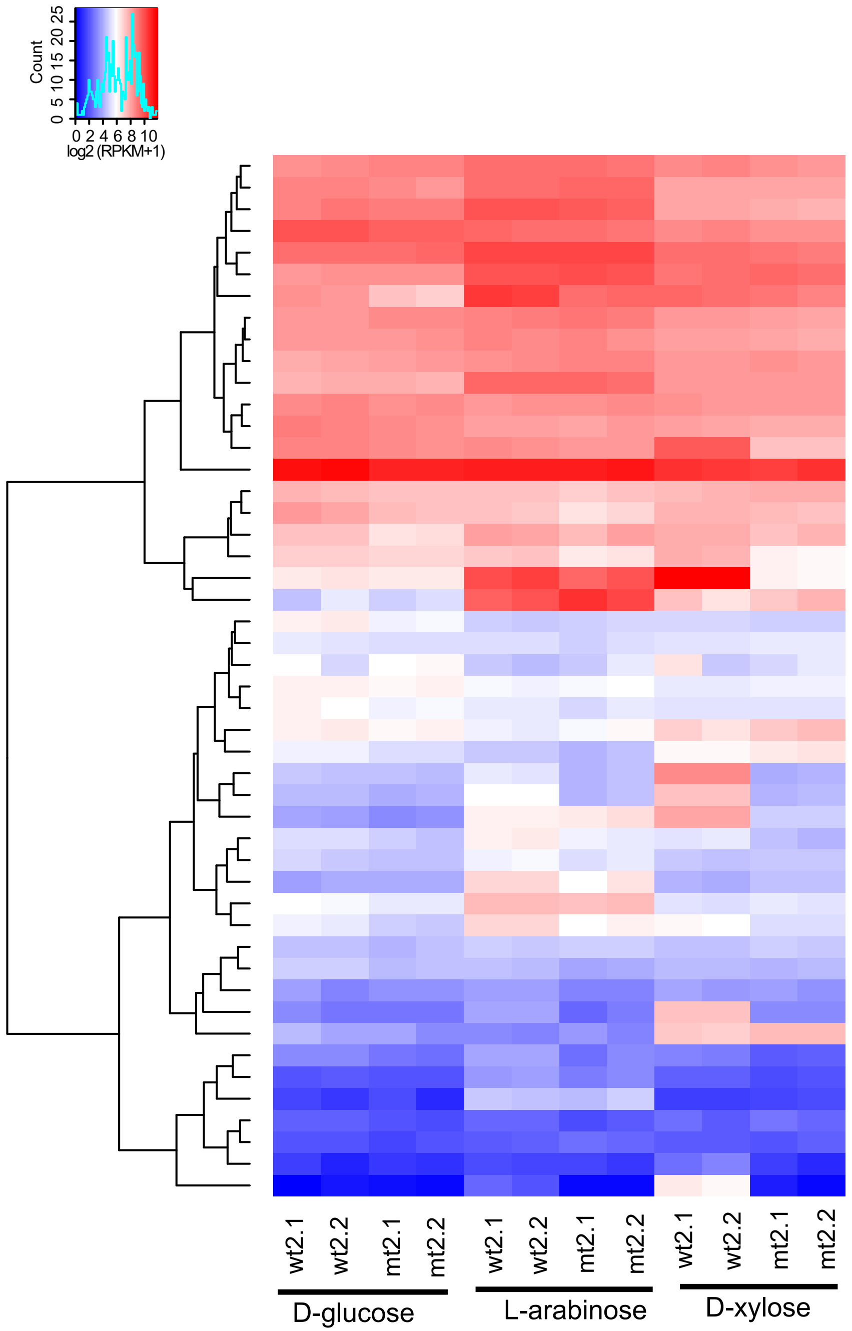

Gene id	Pathway	Gene name in *A. niger*
2297263	glycolysis	
2300033	glycolysis	*fbaA*
2316731	TCA cycle	*aclA*
2295989	glycolysis	*enoA*
2312570	TCA cycle	*citA*
2295514	TCA cycle	*idpA*
2140820	glycolysis	*aldA*
2080603	glycolysis	
2303832	glycolysis	*pda1*
2312383	TCA cycle	*kgdA*
2309210	TCA cycle	*lpd1*
2315052	TCA cycle	*mdhA*
2316240	glycolysis	*pgkA*
2300643	Pentose Phosphate Pathway	*tktA*
2311855	glycolysis	*gpdA*
2294943	mannose	*pmmA*
2300520	glycolysis	
2097501	glycolysis	*acuA*
110022	Pentose Catabolic Pathway/Galacturonic acid	*larA/gaaD*
43671	Pentose Catabolic Pathway	*xyrA*
2315623	glycolysis	
2297503	glycolysis	*pfkA*
2300308	L-rhamnose	
112121	glycolysis	*pdcA*
2303016	mannose	*pmiA*
2296974	Pentose Phosphate Pathway	*rpeA*
2297364	glycolysis	*glkA*
2299590	Leloir pathway	*galE*
2082283	glycolysis	
67060	Pentose Catabolic Pathway	*xkiA*
2293953	Pentose Catabolic Pathway	*xdhA*
55576	glycolysis	*adhA*
82505	Galacturonic acid	*gaaB*
57502	glycolysis	
2302811	Pentose Catabolic Pathway	*lxrA*
112014	glycolysis	
2308351	Pentose Phosphate Pathway	*talB*
2304244	glycolysis	
102290	L-rhamnose	*lraA*
2298452	glycolysis	
84302	glycolysis	
79295	Galacturonic acid	*gaaA*
87557	Galacturonic acid	*gaaC*
62052	Pentose Catabolic Pathway	*ladA*
103080	L-rhamnose	*lraB*
81869	L-rhamnose	*lraC*
39694	glycolysis	*tpiA*
89872	Pentose Phosphate Pathway	*rpiB*

Figure S4. Hierarchical clustering of significantly differentially expressed putative metabolic genes in the Δ*Mtxyr1* strain. The wild type and Δ*Mtxyr1* strain were grown for 2 h on D-glucose, L-arabinose and D-xylose. The color code represents the logged expression values (RPKM+1) of both biological duplicates (named 2.1 and 2.2). Behind the JGI protein numbers, the pathway the gene is putatively involved in is given, while the third column contains the gene name of the ortholog from *A. niger*.
